# Average Longevity

**Published:** 1914-05

**Authors:** 


**Average Longevity.** It is estimated that the length of life, 400 years ago, was about 18 or 20, due to high infant mortality,, wars, epidemics and violence. 100 years ago it was about 30. Now it is about 40 in civilized countries of temperate climate.

